# Energy, Entropy and Quantum Tunneling of Protons and Electrons in Brain Mitochondria: Relation to Mitochondrial Impairment in Aging-Related Human Brain Diseases and Therapeutic Measures

**DOI:** 10.3390/biomedicines9020225

**Published:** 2021-02-22

**Authors:** James P. Bennett, Isaac G. Onyango

**Affiliations:** 1 Neurodegeneration Therapeutics, Charlottesville, VA 22901, USA; 2 International Clinical Research Center, St. Anne’s University Hospital, CZ-65691 Brno, Czech Republic; igonyango2@gmail.com

**Keywords:** mitochondria, electron transport chain, oxidative phosphorylation, ATP, brain energy metabolism, neurodegenerative diseases, oxidative stress, nitrative stress

## Abstract

Adult human brains consume a disproportionate amount of energy substrates (2–3% of body weight; 20–25% of total glucose and oxygen). Adenosine triphosphate (ATP) is a universal energy currency in brains and is produced by oxidative phosphorylation (OXPHOS) using ATP synthase, a nano-rotor powered by the proton gradient generated from proton-coupled electron transfer (PCET) in the multi-complex electron transport chain (ETC). ETC catalysis rates are reduced in brains from humans with neurodegenerative diseases (NDDs). Declines of ETC function in NDDs may result from combinations of nitrative stress (NS)–oxidative stress (OS) damage; mitochondrial and/or nuclear genomic mutations of ETC/OXPHOS genes; epigenetic modifications of ETC/OXPHOS genes; or defects in importation or assembly of ETC/OXPHOS proteins or complexes, respectively; or alterations in mitochondrial dynamics (fusion, fission, mitophagy). Substantial free energy is gained by direct O_2_-mediated oxidation of NADH. Traditional ETC mechanisms require separation between O_2_ and electrons flowing from NADH/FADH_2_ through the ETC. Quantum tunneling of electrons and much larger protons may facilitate this separation. Neuronal death may be viewed as a local increase in entropy requiring constant energy input to avoid. The ATP requirement of the brain may partially be used for avoidance of local entropy increase. Mitochondrial therapeutics seeks to correct deficiencies in ETC and OXPHOS.

## 1. Introduction

A 70 kg human adult nominally makes ~70 kg of adenosine triphosphate (ATP) per 24 h. Under conditions of normal oxygen availability, most of this ATP is made in mitochondria by oxidative phosphorylation (OXPHOS) of energy substrates directly or indirectly created by solar photons through photosynthesis. This amount of ATP (8.31 × 10^25^ molecules/24 h) requires ~20.8 × 10^25^ electrons/24 h to be passed through the mitochondrial electron transport chain (ETC), when ~2.5 electrons are required for each ATP generated by ATP synthase under normal coupling. If the adult brain, which comprises 2–3% of body weight, but consumes at least ~20% of molecular oxygen and energy substrates [1], contains on average 86 billion (86 × 10^9^) neurons [2], and if each neuron contains 1000 mitochondria (likely an overestimate), then each neuronal mitochondrion in the brain must pass on average 1.40 × 10^7^ electrons/s to maintain ATP production. This estimation is based on glucose utilization/oxygen consumption being split 1–1 between neurons and nonneuronal cells in the brain and is not corrected for glial generation of lactate (from glucose) and neuronal metabolism of glial lactate.

If each neuronal mitochondrion has 10^4^ ETC macrocomplexes (“respirasome”; likely an overestimate) along the inner membrane cristae, then each ETC macrocomplex passes ~1400 electrons/s (0.0007 s·electron^−1^) and at least ~7000 protons/s (0.00014 s·proton^−1^) to maintain ATP production. (Note that this estimate of proton translocation rate (1.4 × 10^−4^ s/proton) is comparable to the lower estimate (0.35 × 10^−4^ s) of proton tunneling time through an AT base pair in DNA at room temperature [3,4].) These calculations represent lower-limit averages with debatable assumptions (i.e., likely more protons are translocated), and they do not include corrections for electron or proton leakage/scavenging, variations in coupling between electron flow and ATP synthesis, variations in substrate availability, or other conditions of mitochondrial “health”.

What is apparent from these average estimates of electron and proton velocities (*momenta*, if particle masses are also considered) is that Nature has designed in mitochondria efficient and stereotyped mechanisms for controlling electron and proton flow to transform potential energies of solar photon-derived small molecules acquired by photosynthesis into ATP. One intriguing but unresolved question is whether mitochondria ultimately represent an organelle mediating a transition between quantum and classical behaviors of electrons and protons. Again, note that several-fold more protons are pumped than electrons are passed to maintain ATP production.

The ETC/OXPHOS process in mitochondria is therefore critical to energy metabolism in the brain. The mitochondrial ETC/OXPHOS process is also damaged during human aging, mainly as a result of consuming so much oxygen, leading to “oxidative stress”. Such damage is particularly meaningful for brain energy metabolism and may account for the increased incidence of degenerative brain diseases associated with aging.

## 2. Quantum Tunneling of Protons and Electrons in Mitochondria

Electrons (mass = 9.11 × 10^−28^ gm) and protons (mass = 1.67 × 10^−24^ gm) are both quantum entities that are best described as waves existing in probabilistic vector spaces (“quantum fields”) with “spin” one-half and are either elementary particles (electrons) or composed of quarks (protons) in the standard model. These “waves” become “particles” upon certain types of detection, potentially explaining the wave–particle duality universally observed in quantum entities since their descriptions in the early 20th century (see [5]).

According to the Heisenberg uncertainty principle, the locations and momenta of electrons and protons cannot be precisely known at the same time, at least when moving through empty space. Yet contemporary descriptions of mitochondrial function appear to violate this principle, and it is critical that the momentum-location constraints on electrons and protons be kept in mind as mitochondrial ETC activity is analyzed.

It is likely, though not proven, that proton pumping (mitochondrial proton-coupled electron transfer (PCET)) occurs in Complex I through protein subunits separable from those mediating electron transport [6]. These proton-pumping complexes appear to be composed of the seven hydrophobic Complex I subunits coded by the mitochondrial genome (mtDNA) [6]. If this formulation is correct, then damage/mutations to mtDNA (at least to the seven Complex I subunits) will selectively affect proton pumping rates and not directly alter ETC catalytic rates.

Although the electron acceptor molecule (ubiquinone) for electron flow in Complex I is well characterized, there does not appear to be a separate proton acceptor molecule in the intermembrane space, other than water molecules. Because the downstream ATP synthase rotor head (for OXPHOS) appears to accept only protons (not hydrated protons, [7]), this situation begs the question of how pumped protons are “protected” from hydration by water molecules in the mitochondrial intermembrane space. (Note that proton solvation by water is very energetically favorable with Free energy ~266 kcal/mol.). Perhaps there exists an as of yet unknown proton acceptor molecule in the intermembrane space (other than water) with different thermodynamics of proton binding? An alternative mechanism proposed by Leone, et al. [7] is that the rotor arms of ATP synthase operate using a gradient of un-hydrated protons bound to carboxylate anions, with water molecules separately bound to the rotor arms.

## 3. Decoherence

It is debatable as to whether decoherence occurs to a significant degree in mitochondria. Stated simplistically, decoherence (loss of “quantum-ness”) occurs when there is interaction of quantum entities with non-quantum, classical macroscopic objects such that quantum behavior is reduced or lost [8]. Mitochondria may properly be considered macroscopic entities; whether Complex I iron–sulfur centers with low energy molecular orbitals that are separated by 14 angstroms or less, and thus form “wires” for conducting electrons, meet the same criterion is debatable. The addition of nearby water molecules arranged in tandem appears to provide a pathway for electron tunneling through these wires, which reduces activation energies (thus increasing rates) but theoretically has no effect on the energetics of electron movement ([9] and references therein).

**Figure 1:**
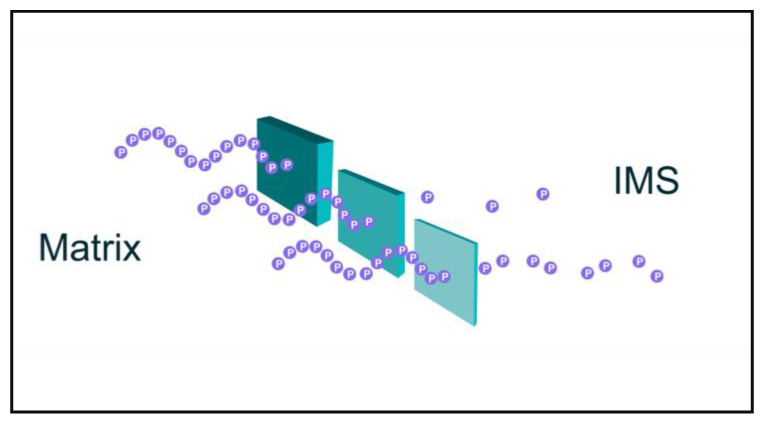
Cartoon of proton tunneling. Protons pumped from the mitochondrial matrix into the intermembrane space (IMS) must overcome electrostatic repulsion of other protons (already in the IMS?) and likely avoid “irreversible” solvation by water. These are but two of likely several energy barriers that protons must overcome, and “quantum tunneling” may provide a mechanism to overcome energy barriers experienced by protons moving into the IMS and increase rates of proton pumping.

Tunneling is the phenomenon of quantum entities appearing to pass through energy barriers due to their wave properties and small (nonzero) probabilities (wave function (Ψ^2^)) of existing on either side of barriers defining an energy well [10] (also, see Figure 1). Tunneling is considered to be a quantum phenomenon; thus, if decoherence is dominant in mitochondrial ETC function, then tunneling is less probable. Contrarily stated, the greater the quantum-ness of ETC behavior, the greater the probability that tunneling may occur.

Shown in Figure 1 are protons (purple spheres) moving as a sine wave through barriers of variable thickness. Mathematically, quantum tunneling may be viewed as follows: Let ***P*** be the probability of a particle with mass ***m*** and energy ***E*** passing through a barrier with energy ***V***:

(1) $$P=\exp\left(\frac{-4\alpha \pi }{h}\sqrt{2m\left(V-E\right)}\right)$$

where ***V*** is the energy of the potential barrier, ***E*** is the kinetic energy possessed by the particle, ***α*** is the thickness of the barrier, ***m*** is mass of the particle (in the case of protons, mass = 1.67 × 10^−24^ gm), and *h* is Plank’s constant (6.626 × 10^−34^ m^2^·kg/s). (Above taken from ChemLibre Texts: https://chem.libretexts.org/Bookshelves/Physical_and_Theoretical_Chemistry_Textbook_Maps/Supplemental_Modules_(Physical_and_Theoretical_Chemistry/Quantum_Mechanics/02._Fundamental_Concepts_of_Quantum_Mechanics/Tunneling (accessed on 21 February 2021)). (The above are taken from [9]).

By this formula, decreasing barrier thickness (***α***) will increase the probability of tunneling through an energy barrier. This is presented in Figure 1.

Electron tunneling in Complex I was recently reviewed [9] (and references therein). Proton tunneling has been described in laboratory experiments, typically performed at very cold temperatures and high pressures [11]. Proton tunneling in mitochondrial ETC has not been described but has been proposed to occur through hydrogen bonds in replicating DNA molecules as a mechanism of spontaneous DNA mutation [3]. Proton tunneling may also occur in the “Grotthuss” mechanism of “proton jumping”, whereby protons move through a hydrogen bond network of adjacent water molecules [12,13].

**Figure 2:**
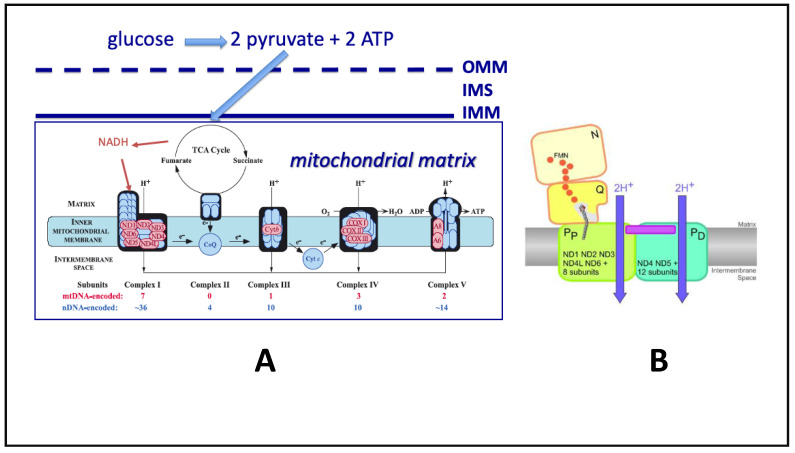
Overview of Mitochondrial Electron Transport Chain ETC/Oxidative Phosphorylation (OXPHOS) and ATP Production. (A). (left) Linear representation of the mitochondrial ETC/OXPHOS system. Shown are the 5 mitochondrial complexes involved in ETC/OXPHOS. In pink are the 13 individual protein subunits derived by transcription of mtDNA genes (usually maternally derived) and translated in the mitochondrial matrix. In blue are the protein subunits derived from nDNA (paternally and maternally derived) that are synthesized in the cytosol and imported into mitochondria. Mammals are now thought to have a total of 45 subunits in Complex I (7 from mtDNA, 38 from nDNA). In the brain, glucose is believed to be the major carbon energy source that is transformed to ATP. In other tissues, mitochondria can metabolize fatty acids and amino acids. Glucose (6 carbons) is broken down outside of mitochondria into 2 pyruvate molecules (3 carbons each) by glycolysis, and the resulting pyruvate is imported into the mitochondrial matrix by specific pyruvate carrier proteins that span the relatively protein-rich outer mitochondrial membrane (OMM), the intermembrane space (IMS), and the relatively lipid-rich inner mitochondrial membrane (IMM). Once in the matrix, pyruvate is oxidatively decarboxylated by the tricarboxylic acid cycle (TCA cycle), yielding reducing electrons that in pairs reduce the electron carrier NAD^+^ to NADH. NADH is subsequently oxidized back to NAD^+^ at Complex I (thus its name, *NADH-NAD oxidoreductase*) and transfers its two electrons to flavin mononucleotide (FMN) that is embedded in the hydrophilic (matrix) arm of Complex I. FMN then passes these two electrons through the Fe–S centers of Complex I to reduce the electron carrier ubiquinone to ubiquinol. This reaction provides the initial free energy that is used for proton pumping. Ubiquinol is reoxidized to ubiquinone at Complex III, where a separate electron carrier in the IMS, cytochrome C, is reduced. Reduced cytochrome C is reoxidized at Complex IV, giving up electrons that participate in the reduction of molecular oxygen to water. Protons are pumped into the IMS at Complexes I, III, and IV (none at Complex II, now regarded as a component of the TCA cycle), and the resulting proton gradient is used to drive ATP production by Complex V. (B). (right) Cartoon showing separate ETC and proton-pumping components of Complex I (taken from Figure 6 of [6]). Shown are the embedded FMN moiety and the nine Fe–S centers that pass electrons through Complex I (orange circles), leading to reduction of ubiquinone to ubiquinol. Additionally shown are the proposed separate proton-pumping subunits of Complex I that consist of proximal (P_P_) and distal (P_D_) subunits that are both believed to be located primarily in the lipid IMM and are in turn composed of the 7 hydrophobic proteins coded by mtDNA and 8 or 12 proteins coded by nDNA, respectively. Proton tunneling into the IMS may occur at these sites.

PCET [14] has been proposed (at least in X-ray resolved crystals of bacterial Complex I) to occur by the combination of electrostatic charge-mediated conformational change in tertiary structure of proton channels following reduction of ubiquinone [15,16]. By this “action at a distance” mechanism, channels in proton-pumping Complex I subunits (see Figure 2) are opened as a result of two electron reduction of ubiquinone following attachment of NADH to its binding site in the matrix side of Complex I and oxidation of NADH to NAD+ with reduction of attached FMN cofactor, followed by passage of the two resulting electrons to ubiquinone to form ubiquinol.

The energetics of ubiquinone reduction theoretically drive the translocation of protons across the inner membrane through proton channels in Complex I subunits “opened” by ubiquinone reduction, but a simpler proton tunneling mechanism may be operative and contribute to the overall PCET rate of Complex I (please also see [6,15,16,17]). Note that the proposal of proton tunneling does not change the bioenergetics, which depend on the free energy of ubiquinone reduction to ubiquinol, but proton tunneling could lower activation energy of proton passage and thus increase the rate of proton pumping. Ubiquinone reduction-induced conformational changes (“action at a distance”) and proton tunneling could synergistically work together to induce Complex I proton displacement, such that proton pumping would not be rate limiting in Complex I PCET. (Please see Figure 2 for a current model of Complex I subunits mediating proton pumping).

Which mechanism might be affected in neurodegenerative diseases (NDDs) (if either mechanism is even operative) is unknown. The possibility should also be considered that proton pumping is not directly affected by NDD pathobiology, and that the major deficit leading to a reduced rate of ATP synthesis is lowering of electron transport rate. Finally, we are aware of no data supporting or refuting the existence of electron quantum tunneling in the other ETC complexes (beyond Complex I, which has been crystallized) and no data supporting or refuting the existence of proton quantum tunneling in any ETC Complex.

## 4. Entropy and Neurodegeneration

Whatever origin of the observable universe hypothesis one subscribes to, all can agree that the cosmological evidence supports the ongoing expansion of the observable universe with a resulting steady increase in entropy. Earlier thermodynamic theorists (such as Clausius) describing the varying forms of this “Second Law of Thermodynamics” developed the concept that the entropy in the Universe may remain constant but is more likely constantly increased, regardless of what happens locally.

The human brain and its ~86 billion neurons display a marked (increase in order)/(decrease in disorder) that represents thermodynamically a decrease in entropy of cellular molecules. In fact, all cells, and life forms themselves, represent a reduction in molecular entropy; from this perspective, cell and organismal death can be viewed as an (inevitable) increase in molecular entropy.

Might neurodegeneration and neuronal death also be viewed as a local increase in entropy, driven ultimately by a reduction in energy input, whatever the “genesis” cause(s) of the bioenergetic deficit? By this paradigm, neuronal death and its attendant increase in molecular entropy would be thermodynamically favored, independent of whether it occurred during “life” or after organismal “death”. If occurring during life, then a clinical phenotype is generated that can be discerned (i.e., loss of cognitive capacity in Alzheimer’s disease (AD); loss of smooth voluntary movement in Parkinson’s disease (PD); loss of muscle mass and appearance of weakness in amyotrophic lateral sclerosis (ALS), etc.).

If this paradigm is true, then prevention of neuronal death (in NDDs) can be viewed as a thermodynamic problem with potential thermodynamic solutions. For example, energy input, which is already disproportionately elevated in adult human brain, could be increased by processes that stimulate mitochondrial energy transformation and ATP synthesis. One could also attempt to increase synaptogenesis and size/interactions of neuronal networks (which should also reduce neuronal molecular entropy). However, we wish to note that no reported therapeutic strategies derived from the above thermodynamic hypothesis of neuronal death have yet been published.

## 5. Oxidative Phosphorylation (OXPHOS) Alterations in NDDs

OXPHOS is an evolved process in which electron flow through the ETC is coupled to proton translocation from the mitochondrial matrix to the intermembrane space, creating a proton and pH gradient between the mitochondrial matrix and intermembrane space. The resulting proton gradient is used to rotate the arm of ATP synthase, an evolutionarily old enzyme [18] that appears to require non-hydrated protons to operate [7] (see above). As discussed previously, this PCET may utilize proton tunneling and/or structural alterations in proton-pumping subunits of the ETC (at least for Complex I).

Because electron flow (at least in Complex I) is believed to use a tunneling mechanism (for discussion see [9] and references therein), structural alterations to proteins critical to electron tunneling may result in reduced rates of electron flow, leading to reduced rates of proton pumping and ATP synthesis. This could result in a bioenergetic deficiency state, based on maintaining a minimum rate of ATP synthesis necessary for neuronal functions (see above). By this mechanism, proton pumping (PCET) would not be mechanistically impaired per se, just reduced in rate.

In NDDs variable reductions in ETC rates at one or more specific complexes have been described. Epigenetic modifications potentially responsible for these reductions include pre-transcriptional changes to genes such as gene methylation and histone modifications that affect gene promoter or repressor activities. Epigenetic alterations have been described in amyotrophic lateral sclerosis (ALS, [19]), Parkinson’s disease (PD, [20,21,22,23,24,25,26,27,28]), and Alzheimer’s disease (AD, [19,20,21,22,25,28,29,30,31,32,33,34,35]). In many studies, cell or animal models of NDDs are utilized, with the understanding that similar phenomena may occur in the more common sporadic forms of each NDD.

Reductions in bioenergetics may also derive from nitrative damage to proteins, particularly to nitration of tyrosine residues by peroxynitrite anion (ONOO^−^). So-called “nitrative stress”, which is frequently found in models that demonstrate “oxidative stress”, have been described in ALS [36,37,38,39,40,41,42,43], AD [36,37,40,44,45,46,47,48,49,50,51,52,53,54,55], and PD [36,37,40,42,44,53,55,56,57,58,59,60,61,62,63,64,65,66,67,68,69,70,71,72,73] tissues.

Oxidative stress (OS) is the condition where production rates of oxidizing species exceed rates of inactivation. Oxidizing species may damage lipids, nucleic acids, and proteins and thus are potentially toxic to cells and energy production at several levels. Because most molecular oxygen is utilized by mitochondria for ETC activity (and is reduced to water), mitochondria are particularly susceptible to OS. OS damage has been described in ALS [74,75], AD [31,35,44,45,50,52,53,55,76,77,78,79,80,81,82,83,84,85,86,87,88,89], and PD [24,36,53,55,56,57,60,62,63,64,65,66,67,71,77,90,91,92,93,94,95,96,97] tissues and models.

## 6. Summary of ETC-OXPHOS

The human brain has disproportionately elevated (~10-fold, relative to mass) energy substrate and oxygen consumption rates. These elevated metabolic rates in brain likely depend on electron and proton tunneling in mitochondria, although neither of these processes has been conclusively demonstrated to occur. Mitochondria can be viewed as transitional organelles that bridge the quantum world of very small wave-particle behavior and the classical world of decoherent larger, more macroscopic structures such as cells.

Photosynthesis yields both the small molecules that directly or indirectly drive mitochondrial electron transport and the toxic by-product (molecular oxygen) that is an excellent electron acceptor (oxidant) for terrestrial life. The highly electrophilic nature of molecular oxygen requires “protection” of reducing equivalents (as mainly NADH) and competes with ETC thermodynamics to yield oxygen free radicals. These free radicals must be detoxified or they will damage cellular constituents (proteins, lipids, nucleic acids) and can combine in several ways with other molecules to yield nitrogen–oxygen toxins (“nitrative stress”).

Protons pumped into the intermembrane space theoretically require protection from thermodynamically favorable hydration, since non-hydrated protons appear to be favored for driving the ATP synthase rotor [7]. How this occurs is presently unknown but may require anatomic proximity of ATP synthase rotor proton-binding sites to proton-pumping sites or an as yet unknown proton solvation system other than water alone. An alternative mechanism presented by Leone et al., involves carboxylate protonation (by non-hydrated protons) and binding of water (from hydronium ions) to ATP synthase [7]. By this mechanism, hydrated protons could drive ATP synthase, but the H_3_O^+^ ions would dissociate rapidly into H^+^ and H_2_O that would separately bind to ATP synthase.

These massive energy transformation systems appear to be damaged in neurodegenerative diseases (NDDs), at least in terms of ease of detecting epigenetic alterations/oxidative stress damage/nitrative stress damage. The result could be a reduction in neuronal ATP synthesis rate, with neuronal dysfunction leading to emergence of early clinical phenotypes and an ultimate increase in entropy following neuronal death or even autophagic digestion of organelles (i.e., mitophagy).

Nature was tasked with producing large quantities of ATP that are used by neurons for many purposes, including the lifetime (usually over many decades) maintenance of nondividing state and recharging of neuronal potentials where rapid potential swings are necessary for functions of both individual neurons and neuronal networks. A truly remarkable system resulted, which appears to fade as organisms age and accumulate biochemical damages over a lifetime. Whether these aging phenomena can be more successfully controlled and the burden of NDD reduced are future challenges to mitochondrial therapeutics.

## 7. Brain Mitochondrial Therapeutics

The human brain is one of several tissues that are “non-mitotic”, meaning that the majority of its cells do not undergo cell division during most of the organism’s lifetime. In fact, entering the cell cycle is considered a lethal event for mature neurons, compared to “mitotic” cells of mesodermal and endodermal origins that regularly die, divide, and are thus replaced.

Even in non-mitotic neurons, mitochondria undergo their own cycles of DNA (mitochondrial DNA, mtDNA) replication. While the mechanistic details of mtDNA replication remain debated, all agree that mtDNA replication is independent of host cell division in both non-mitotic (should be small or nonexistent) and mitotic tissues.

We have yet to learn how mtDNA replication is regulated, although some knowledge exists about the molecules and their hierarchy of control for mtDNA replication. For instance, mtDNA replication utilizes a DNA polymerase specially synthesized by nuclear genes for mtDNA replication, coded for by host cells (DNA polymerase gamma) and imported into mitochondria. There appear to be multiple copies of mtDNA within each mitochondrion, but it remains unclear how that number is regulated. Additionally, not all copies of mtDNA within each mitochondrion, and thus within each cell, are necessarily identical, a condition known as heteroplasmy. In addition, mtDNA appears to have a higher mutation rate than does nuclear DNA, ascribed to both the relative lack of protective proteins and limited DNA repair mechanisms.

The thirteen genes encoded by mtDNA (all for ETC/OXPHOS function) are believed to be translated within the mitochondrial matrix using a genetic code similar to but not identical with the code used in nuclear DNA–nuclear mRNA translation. Special mitochondrial chaperone proteins (again provided by the host cell) appear to assist assembly of the ETC/OXPHOS complexes that are characterized by many nuclear DNA-encoded subunits and lesser numbers of more hydrophobic mtDNA-encoded subunits. Again, several of the regulatory proteins for mtDNA transcription (synthesized from nuclear DNA genes and imported into mitochondria) are known, but the complete details of regulation of mtDNA transcription and translation/assembly into functioning ETC/OXPHOS complexes remain unclear.

Mitochondrial therapeutics strategies, in terms of ATP production, are difficult to implement currently, due mainly to ignorance about details of how mitochondria within brain neurons (and many other cell types) regulate/are regulated in terms of ETC/OXPHOS and thus ATP production capacities. Several approaches can be discussed, and this list is by no means complete:

- Correction of mtDNA mutations
- Correction of mtRNA and/or mitochondrial mRNA errors
- Increase in mitochondrial mass leading to increased ETC/OXPHOS capacity to make ATP
- Prevention of epigenetic, nitrative stress (NS) and oxidative stress (OS) damage to ETC/OXPHOS genes or proteins

The above potential strategies relate solely to mitochondrial ETC/OXPHOS function and not directly to regulation of mitochondrial calcium signaling or cell death initiation, important mitochondrial functions not addressed in this review.

### 7.1. Correction of mtDNA Mutations

The development of rapid and relatively inexpensive “next-generation” DNA sequencing has allowed the development of “3-parent babies” as a viable strategy for prevention of mtDNA-transmitted mutations. If precautions are taken to screen out mitochondrial “pseudogenes” (stretches of nuclear DNA containing variable amounts of mtDNA sequences [98]), then specific mtDNA mutations can be defined in oocytes of mothers who have given birth to a child with a mtDNA mutation-derived disease. Because the mother’s oocytes may contain variable proportions of mutant compared to wild-type mtDNA (recall heteroplasmy), and because maternal transmission of mtDNA is the rule, implantation and growth of oocytes containing only wild-type mtDNA and both maternal and paternal nuclear genomes is now possible with mitochondrial replacement therapy. This can be accomplished by transferring the maternal meiotic nuclear spindle into a donor oocyte that contains only wild-type mtDNA (and has its own nuclear meiotic spindle removed), followed by fertilization with paternal sperm. This technique is referred to as maternal spindle transfer (MST). An alternative approach is to fertilize a donor oocyte with paternal sperm, then remove the paternal-donor pronuclei and replace them with pre-fusion maternal and paternal pronuclei. This approach is known as pronuclei transfer (PNT). See [99] for details. These approaches to a 3-parent baby have been developed, discussed, and implemented in the UK by the Newcastle group [99,100,101,102,103,104] and by the Mitalipov group at Oregon Health Sciences University [100,105,106,107,108,109,110,111,112,113,114,115,116,117,118,119].

### 7.2. Correction of mtRNA and/or Mitochondrial mRNA Errors

The mtDNA genome contains 13 sequences/genes for ETC/OXPHOS proteins that are made in the mitochondrial matrix, 22 tRNA sequences for ribosomal protein synthesis, and 2 rRNA’s that assist in making ETC/OXPHOS proteins from mtDNA genome sequences (that first must be transcribed into mtRNAs). Many of the mtDNA mutational errors that impact translation of mtRNA sequences are mutations in one or more of the tRNA genes in circular mtDNA [120,121]. In addition, it remains unclear how mitochondria maintain an adequate supply of tRNAs needed for synthesis of multiple proteins [122]. Post-transcriptional mt-tRNA gene modifications may also play a role in mitochondrial RNA-based diseases [123].

There are several published reports of correcting mt-tRNA mutation defects, usually by rescuing the respiratory phenotypes of cells harboring specific mt-tRNA mutations (for example, see [124]). These are successful but appear to be restricted to specific mutations, although a more “generic” approach has been reported [125]. This approach utilizes the mitochondrial importation of wild-type tRNAs fused with a mitochondrial importation signal. Using this approach, the authors were able to partially correct metabolic abnormalities of cybrid cells carrying mutations for MELAS (mitochondrial encephalopathy lactic acidosis and stroke) or MERRF (mitochondrial encephalopathy and ragged red fiber disease) [125].

### 7.3. Increase in Mitochondrial Mass Leading to Increased ETC/OXPHOS Capacity to Make ATP

Mitochondrial mass is controlled by the processes of mitochondrial biogenesis (also known as mitobiogenesis, increases mitochondrial mass) and mitochondrial autophagy (also known as mitophagy, decreases mitochondrial mass). Both processes are important for maintaining overall neuronal and cellular bioenergetic function, are operative under normal circumstances, and can be impaired in certain disease phenotypes. Several key pathways are known for mitobiogenesis [126,127,128,129,130,131,132,133,134,135,136,137,138,139,140,141,142,143,144,145,146,147,148,149,150,151,152,153,154,155,156,157,158,159,160,161,162,163,164,165,166,167,168,169,170,171,172,173,174,175,176,177,178,179,180,181,182,183,184,185,186,187,188,189,190,191,192,193,194,195,196,197,198,199,200,201,202,203,204,205,206,207,208,209,210,211,212,213,214,215,216,217,218] and mitophagy [56,61,64,93,139,145,151,163,192,194,195,205,209,213,219,220,221,222,223,224,225,226,227,228,229,230,231,232,233,234,235,236,237,238,239,240,241,242,243,244,245,246,247,248]. The reader is directed to comprehensive reviews of these two important subjects (for mitobiogenesis: [129,130,136,164,167,192,193], and [211]; for mitophagy: [226,233], and [243]).

An obvious question relates to increasing mass of mitochondria containing damaged mtDNA. There are at least two related issues to consider. First, increasing mass of impaired mtDNA-containing mitochondria may improve bioenergetics for the host cell, which is a desired therapeutic goal. The same argument can be applied to cells containing mutated nuclear DNA coding for mitochondrial genes. Second, it remains unclear whether stimulation of mitobiogenesis (or manipulation of mitophagy) will yield net positive or negative effects on cells harboring a heterogenous collection of mitochondria. It must be remembered that effects on peripheral tissues do not necessarily extend into brain tissues.

It is likely that such experiments will need to be tested on an individual’s cells before being applied to that individual. Stimulating mitobiogenesis or manipulating mitophagy could be performed on white blood cells or muscle cells, both readily accessible tissues that are mitotic and non-mitotic, respectively. Improvements in respiration or ATP synthesis rates can be assayed in response to several agents.

### 7.4. Prevention of Epigenetic, Nitrative Stress (NS) and Oxidative Stress (OS) Damage to ETC/OXPHOS Genes (Epigenetics) or Proteins (NS and OS)

This final approach represents decades of investigation by many scientists, is potentially applicable to both specific clinical phenotypes and the broad area of aging, and likely will continue to be popular in the future. Mitochondrial respiration, particularly in brain neurons and generally throughout the body, suffers from taking place in environments with relatively high levels of oxygen molecules and oxygen–nitrogen adducts (such as peroxynitrite anion, ONOO-), or nitric oxide (NO) itself). In addition, it remains unclear how the > 80 genes responsible for mitochondrial proteins of the ETC and OXPHOS systems are regulated by epigenetics, but recent studies suggest that this does occur and that mitochondrial metabolism can affect nuclear epigenetics [107,249,250,251,252].

There have been many attempts to develop therapies directed toward reduction of OS and/or NS. The most promising utilize molecules that are either organic cations at physiological pH, such as pramipexole [253,254], or are attached to “inactive” organic cationic groups such as triphenylphosphonium (TPP) [255,256,257,258] or rhodamine [256,259,260,261,262]. The underlying concepts are that by virtue of lipophilicity, such potential therapeutics can pass through cell and mitochondrial membranes, and the cationic nature suggests that such molecules will be concentrated into the relatively negative mitochondrial matrix (a result of proton pumping).

The OS/NS scavenging molecules must have intrinsic activity and their concentration into the mitochondrial matrix adds organelle specificity. It is striking that the capacity of mitochondrially-targeted ROS and RNS ultimately derive from proton pumping across the inner membrane (responsible for the mitochondria membrane potential), which may involve several mechanisms of PCET, including proton tunneling.

Both NS and OS have been reduced in brain and specifically human disease models [39,50,52,53,55,56,67,76,78,80,84,91,94,95,96,257,262] by such approaches. This therapeutic area appears to be popular in attempting to improve mitochondrial bioenergetics in nervous tissues, as well as other organs.

## 8. Conclusions

Mitochondria have evolved, likely from protobacterial precursors through endosymbiosis [263], and now inhabit cells of almost all terrestrial and marine plants and animals, including humans. In addition to their critical roles in modulating cellular calcium signaling and cell death initiation, mitochondria through ETC/OXPHOS appear to supply most of the substantial daily ATP requirement for humans. Adult human brain has a ~10-fold disproportionate (relative to mass) ATP production rate and depends on the stereotyped movement of reducing electrons down an energy gradient in the ETC and conservation of this ETC energy decrease by proton displacement across the mitochondrial inner membrane. This electron movement and proton displacement, however they occur, must respect quantum mechanical constraints.

Both electrons moving through the ETC and proton displacement from the matrix to the intermembrane space (IMS) may utilize quantum tunneling in addition to other mechanisms. It is not yet clear whether tunneling occurs at all, but it is a theoretically appealing mechanism for quantum entities to pass through energy barriers and reduce activation energies (thus increasing rates of proton transfer).

Mitochondria must likely segregate electrons from electrophilic molecular oxygen and protons from solvation by water. How these feats are accomplished remains unclear, but our daily ATP requirements likely require these biochemical gymnastics. Mitochondria may represent a necessary transitional organelle between the quantum world of elementary particles and energy-releasing catabolism of molecules created from absorbed solar-derived photons. Decoherence (loss of quantum-ness) may assist ATP production in mitochondria, and theoretically its presence may vary with energy needs.

Many neurodegenerative diseases (NDDs) afflicting humans may be viewed thermodynamically as increases in molecular entropy during life of the organism as neurons die. Such local entropy increases may arise from decreased neuronal energy production traceable to decline of OXPHOS rates. OXPHOS rates in turn depend on availability of intact ATP synthase complexes and (likely) non-hydrated protons in the intermembrane space or at the proton-binding sites of the ATP synthase rotor.

Mitochondrial therapeutics can address bioenergetic deficiencies at multiple levels, from epigenetic changes in mitochondrial and/or nuclear genomes, through measures to reduce post-translational damage to ETC/OXPHOS proteins. Many such approaches have been/are being developed, and optimism exists for varied solutions to the human problems of mitochondrial ETC/OP dysfunction in NDDs.
